# Fermentation Versus Meat Preservatives to Extend the Shelf Life of Mealworm (*Tenebrio molitor*) Paste for Feed and Food Applications

**DOI:** 10.3389/fmicb.2020.01510

**Published:** 2020-07-15

**Authors:** An Borremans, Ruben Smets, Leen Van Campenhout

**Affiliations:** Faculty of Engineering Technology, Department of Microbial and Molecular Systems (M2S), KU Leuven, Geel, Belgium

**Keywords:** mealworms, microbial stability, chemical stability, preservatives, fermentation

## Abstract

Freshly prepared pastes from blanched mealworms (*Tenebrio molitor*) are highly perishable and prone to microbial and chemical changes upon storage due to their high water activity, near–neutral pH, and their rich nutrient profile. Their shelf life is short unless preservation methods are used. In this study, the effects of preservatives (sodium nitrite and sodium lactate) and lactic acid fermentation (with the starter cultures Bactoferm^®^ F-LC and *Lactobacillus farciminis*) on the microbiological and the chemical stability of mealworm pastes stored at 4°C were compared. During the storage experiment, the pH, water activity, color, microbial counts, and fat oxidation were monitored. In addition, the prevalence of the pathogens *Bacillus cereus*, *Salmonella* spp., and *Listeria monocytogenes* were studied. Microbial quality evaluation of the mealworm pastes showed that the addition of preservatives did not inhibit microbial growth during refrigerated storage, reaching the upper limits for consumption between seven and 14 days. By contrast, the acid medium (pH < 4.50) created by fermentation stabilized all microbial populations investigated, indicating that these pastes could be consumed up to (at least) 8 weeks of refrigerated storage. *L. monocytogenes*, *Salmonella*, and *B. cereus* were not detected in any of the samples and lipid oxidation of the samples was minimal. Altogether, this study shows that lactic acid fermentation can be used successfully to inhibit microbial growth, to maintain chemical quality, and to extend the shelf life of mealworm pastes stored at 4°C.

## Introduction

Edible insects such as the yellow mealworm [*Tenebrio molitor* Linnaeus (Coleoptera: Tenebrionidae)] are valuable alternatives to conventional meat, because their nutrient content is comparable, and the ecological impact of insect rearing is lower than that of traditional animal husbandry (Oonincx and de Boer, 2012; van Huis, 2015). In some EU member states, such as Belgium and The Netherlands, a variety of food products containing mealworms has been launched on the market since they were authorized for human consumption at national level. Currently, these products can stay on the market at least until their submitted Novel Food dossiers have been evaluated [Regulation (EU) 2015/2283]. Mealworms are incorporated in these products either as whole insects in fresh or dried form, or processed into a powder or paste, or as an extract of protein, fat or chitin to increase their nutritional value or functionality. The use of mealworms as aquafeed and petfood ingredient is already allowed in Europe and they may be authorized in feed for other animal species in the future (International Platform of Insects for Food and Feed [IPIFF], 2019). Several European pet food companies incorporate dried mealworms in their feed formula as a mean to diversify their products’ range or to replace soybean and fishmeal. However, fresh mealworms are perishable. After harvesting and killing, they have a short shelf-life because of microbial growth, lipid oxidation, Maillard reactions, and/or enzymatic reactions (Jeon et al., 2016; Lenaerts et al., 2018; Melgar-Lalanne et al., 2019). These phenomena can cause rancidity with off-flavors, color alterations, and/or loss of nutrients (Chan et al., 1993). In addition, food-borne pathogens may compromise microbial safety. Therefore, to maintain quality and safety of insects and derived products, fresh edible insects need to be stabilized.

Freezing and freeze-drying are the most commonly used methods to convert fresh insects to a storable form after harvesting (Efsa Scientific Committee, 2015). However, both conservation strategies impose drawbacks. Frozen storage implies frozen transport, which adds to the cost price. Moreover, when thawing is not performed correctly (too long and/or no refrigeration after thawing), food safety problems are likely to occur. Freeze-dried mealworms, on the other hand, are chemically and microbiologically stable, but freeze-drying is expensive and also preserves the microbiota, which may cause food safety problems upon processing of the larvae into food products (FASFC, 2018). Also, the brittle texture of freeze-dried larvae limits their applications. The feed and food industries are aware of these restrictions and therefore other preservation technologies have to be explored.

Refrigeration is the best method to retain the sensorial properties of fresh insects. However, this method can only be used to store (already to some extend processed) mealworms for a few days. According to Borremans et al. (2018), blanched mealworms can be stored refrigerated for ten to 12 days without exceeding the general food spoilage level for foods of 7.0 log cfu/g (Sperber and Doyle, 2009). De Smet et al. (2019) observed substantial microbial growth but limited fat oxidation in pastes prepared from steamed mealworms. These mealworm pastes were stored for three weeks at 4°C but were unacceptable for consumption within 14 days. The addition of meat preservatives (sodium nitrite and sodium lactate) did not improve the shelf life of these pastes.

Previous work has shown that a paste produced from blanched mealworms can be fermented using commercial meat starter cultures (Borremans et al., 2019). It was not yet investigated so far whether the shelf life of these pastes can be improved by fermentation and whether the pH can be reduced sufficiently and fast enough by fermentation to prevent spoilage organisms and pathogens from growing (Özogul and Hamed, 2018). Fermentation was demonstrated to extend the shelf life of shrimp paste for weeks or even months (Fagbenro, 1996). In this study, the impact of fermentation on the shelf-life of pastes produced from blanched mealworms was investigated. The shelf life was compared with that of unfermented pastes and unfermented pastes containing typical meat preservatives being sodium nitrite and sodium lactate.

## Materials and Methods

### Preparation of Mealworm Samples

Living mealworms (Nusect, Ledegem, Belgium) were killed by freezing and stored at −18°C until further use. Fermented mealworm samples were prepared as described before (Borremans et al., 2018, 2019) without the addition of the curing agent sodium nitrite. Briefly, 7.0 kg of frozen larvae were blanched (40 s), and mixed into a paste using a kitchen mixer (Bosch CNHR 25). One half of the paste was inoculated with the commercial starter culture Bactoferm^®^ F-LC (Chr. Hansen Holding A/S, Hoersholm, Denmark, 501091, consisting of a mixture of *Pediococcus acidilactici*, *Lactobacillus curvatus*, and *Staphylococcus xylosus*), according to the manufacturers’ instruction (25 *g*/100 kg). The other half was inoculated with the pure culture *L. farciminis* (Chr. Hansen Holding A/S, Hoersholm, Denmark, 501167) to reach a level of ±6.5 log cfu/g paste. The ability of these two starter cultures to ferment a mealworm paste produced at laboratory scale was demonstrated in previous work (Borremans et al., 2018, 2019). Similar to raw meat fermentations, 2.8% NaCl (w/w), and 0.75% d(+)-glucose (w/w) were added to both pastes to provide flavor, to control the background microbiota, and to fuel the fermentation as the fermentable sugar content of mealworms is low (Morales-Ramos et al., 2016). After thorough mixing, the pastes were distributed over sterile 50 ml Falcon tubes (Sarstedt, Antwerp, Belgium) and incubated at 35°C for seven days. After seven days, non-fermented mealworm pastes were prepared (so that their storage period could start together with that of the fermented pastes) by blanching 5.0 kg frozen larvae (40 s) and mixing them into a paste using the same kitchen mixer. An aliquot of 1.5 kg of freshly prepared paste was supplemented with 150 mg NaNO_2_/kg paste (EMSURE^®^, ACS, Reag. Ph. Eur. Analytical reagent, Merck Millipore, Overijse, Belgium), an equal amount was supplemented with 50 *g*/kg paste of a 60% sodium DL-lactate solution (Syrup, 60% w/w, synthetics, Sigma Aldrich, Overijse, Belgium), and the remaining paste remained without preservatives (control). After thorough mixing each type of paste, the pastes were also distributed over sterile 50 ml Falcon tubes (Sarstedt, Antwerp, Belgium) which were completely stuffed. The approach in this study differed from that used by De Smet et al. (2019) who also determined the shelf life of mealworm pastes: in our study, the mealworms were blanched instead of steamed, the mealworm pastes were stored in smaller volumes (50 mL versus 250 mL), the storage period for unfermented pastes was four (rather than three) weeks, and in particular fermented samples were included in the storage period [not in De Smet et al. (2019)].

### Storage Conditions and Sampling Plan

All samples were stored at 4 ± 1°C in a home type refrigerator (Miele, Belgium). Immediately after processing as well as during storage, pH, water activity, color, and microbial counts were monitored. Unfermented samples were analyzed weekly during four weeks after production, while the fermented samples had an additional sampling point after eight weeks of storage. Per sampling time point, three Falcon tubes per condition were withdrawn with each analysis performed once per Falcon tube. In addition, fat oxidation and a selection of food pathogens were monitored after processing and after four and eight (for the fermented samples) weeks of storage. For both pathogen detection and fat oxidation, the contents of six Falcon tubes were mixed and all analyses were performed in triplicate.

### pH, Water Activity and Color Parameters

The pH was measured using a digital pH-meter (Portamess 911, Knick, Germany) equipped with a SI analytics electrode (Germany). The water activity (a_w_) was determined using a LabMaster a_w_, Novasina (Lachen, Switzerland) as described by Wynants et al. (2018) and a colorimeter (CR-5 Konica Minolta) was used to determine the color of the pastes in the CIELAB space. Browning indices, characterizing the intensity of the brown color, and total color differences, using the initial color of the pastes as reference, were determined using the methods described by De Smet et al. (2019).

### Microbial Analyses and Pathogen Detection

The microbiological quality of the mealworm pastes during cold storage was monitored assessing total (an)aerobic bacteria, lactic acid bacteria (LAB), psychrotrophic bacteria (PSY), yeast and molds (Y&M), Enterobacteriaceae (ENT), sulphite reducing clostridia (SRC), and (an)aerobic bacterial spores as described by Borremans et al. (2019), Vandeweyer et al. (2017b), and Wynants et al. (2019).

The prevalence of *Bacillus cereus, Salmonella* spp., and *Listeria monocytogenes* was studied according to ISO 7932 (plate counts), ISO 6579 (absence in 25 *g*), and the ISO method AFNOR BRD 07/4-09/98 (absence in 25 *g*), respectively.

### Fat Oxidation

To analyze fat oxidation, fat was extracted from the pastes following a modification of the procedure proposed by Folch et al. (1957). Briefly, 70 *g* of paste was mixed with 150 mL chloroform/methanol mixture (2:1 v/v, VWR) solution, shaken for 5 min, and then filtered. The solid phase was extracted two more times with 50 mL of the chloroform/methanol mixture. The obtained filtrates were pooled and washed twice with 50 mL of a 0.88% sodium chloride solution. After phase separation, the organic phase was dried using anhydrous magnesium sulfate (VWR) after which the solvent was removed using a rotary evaporator (Büchi). The fat extracts were stored at −18°C until further analysis.

The fat obtained by the Folch method was used to determine the peroxide and *p*-anisidine values. Peroxide values, expressed as units of meq. O_2_/kg fat sample, were determined using the modified method of Wu and Mao (2008) as described by Lenaerts et al. (2018). *p*-anisidine values were determined using the method described by Tenyang et al. (2017) and Lenaerts et al. (2018).

### Statistical Analysis

One-way ANOVA was performed to check whether there were any significant differences between the pH, a_w_, color parameters, microbial numbers, and peroxide and *p*-anisidine values of the different samples during storage. When significant (*p* < 0.05), *post hoc* tests with the Tukey adjustment were performed. All analyses were performed using IBM SPSS Statistics 23.

## Results and Discussion

### pH, Water Activity and Color During Storage

Changes in pH, water activity (a_w_), and color parameters during the refrigerated storage of the pastes are shown in Table 1. Freshly prepared mealworm pastes were characterized by a near-neutral pH (6.54) and high a_w_ values (1.003), which were in line with the findings of Vandeweyer et al. (2017a, b), Lenaerts et al. (2018), and De Smet et al. (2019) for fresh and blanched mealworms and pastes prepared from steamed mealworms. As expected, the pH and a_w_ values of the fermented pastes were significantly (*p* < 0.05) lower than those of the non-fermented pastes at the start of the storage period (day zero), with values ranging from 4.35 to 4.51 and from 0.964 to 0.968, respectively. The decrease in pH is a desirable effect of the metabolic activity of the added starter cultures (Bintsis, 2018). The reduction in a_w_ can be attributed to the addition of D(+)-Glucose (0.75%, w/w) and sodium chloride (2.8%, w/w) to the pastes prior to fermentation. During the first week of storage, the pH of the control and the fermented pastes significantly (*p* < 0.05) decreased and increased, respectively, but stabilized thereafter until the end of storage. The addition of the preservatives sodium nitrite and sodium lactate to the mealworm pastes had no significant effect (*p* < 0.05) on the pH during the entire storage period. This last result is in contrast to the observations of De Smet et al. (2019), where neither of the two preservatives could prevent a reduction in pH during the refrigerated storage of pastes prepared from steamed mealworms. This difference can likely be explained by the fact that prior to the production of the pastes in the latter study, the mealworms were steamed and not blanched. The microbial reduction caused by steaming is lower than that obtained by blanching as demonstrated by De Smet et al. (2019). A higher microbial load remaining after heat treatment can cause the mealworms to spoil faster with a resulting decrease in pH due to metabolic activity, i.e., organic acid production.

**TABLE 1:** Means ± standard deviation (*n* = 3) of pH, a_w_, browning index (BI), and total color difference with the initial value (ΔE) during storage of pastes produced from blanched mealworms (*Tenebrio molitor*) without additive (control), with the additives sodium nitrite or sodium lactate, or fermented with the starter culture Bactoferm^®^ F-LC or *Lactobacillus farciminis*.

Sample	Sampling day	pH	a_w_	BI [-]	ΔE
Control	0	6.54 ± 0.02^a,A^	1.00 ± 0.00^a,A^	36.35 ± 0.37^a,A^	–
	7	5.88 ± 0.03^b,A^	1.00 ± 0.00^a,A^	38.27 ± 0.56^a,b,A^	4.27 ± 0.12^a,b^
	14	6.09 ± 0.25^b,A^	1.00 ± 0.01^a,A,B^	38.11 ± 0.88^a,b,A^	3.63 ± 0.40^b,c^
	21	5.96 ± 0.03^b,A^	1.00 ± 0.01^a,A,B^	40.64 ± 0.40^b,A^	5.19 ± 0.21^a^
	28	6.09 ± 0.13^b,A^	1.00 ± 0.00^a,A^	37.29 ± 1.76^a,A,B^	2.73 ± 0.57^c^
Sodium nitrite	0	6.54 ± 0.02^a,A^	1.00 ± 0.00^a,A^	36.35 ± 0.37^a,A^	–
	7	6.65 ± 0.01^a,B^	1.01 ± 0.01^a,A^	32.46 ± 0.24^b,B^	1.33 ± 0.04^a^
	14	6.59 ± 0.08^a,B^	1.00 ± 0.00^a,B^	32.26 ± 0.09^b,c,B^	1.40 ± 0.09^a^
	21	6.62 ± 0.04^a,B^	1.01 ± 0.00^a,B^	31.75 ± 0.09^c,d,B^	1.60 ± 0.08^a^
	28	6.38 ± 0.29^a,A,B^	1.00 ± 0.00^a,A^	31.47 ± 0.30^d,C^	1.71 ± 0.04^a^
Sodium lactate	0	6.54 ± 0.02^a,A^	1.00 ± 0.00^a,A^	36.35 ± 0.37^a,A^	–
	7	6.64 ± 0.01^a,B^	0.99 ± 0.01^a,B^	32.87 ± 0.38^b,c,B^	1.24 ± 0.19^a^
	14	6.49 ± 0.23^a,B^	0.99 ± 0.01^a,A^	32.59 ± 0.33^c,B^	1.51 ± 0.09^a,b^
	21	6.49 ± 0.2^a,B^	0.99 ± 0.01^a,A^	32.92 ± 0.17^b,c,C^	1.31 ± 0.14^a^
	28	6.61 ± 0.03^a,B^	0.99 ± 0.01^a,A^	33.43 ± 0.09^b,C,D^	1.75 ± 0.08^b^
Bactoferm^®^ F-LC	0	4.50 ± 0.01^a,B^	0.97 ± 0.00^a,B^	37.50 ± 0.47^a,B^	–
	7	4.60 ± 0.04^b,C^	0.97 ± 0.00^a,C^	39.96 ± 0.16^b,C^	0.89 ± 0.14^a^
	14	4.57 ± 0.01^b,C^	0.97 ± 0.00^a,C^	38.96 ± 0.57^b,c,C^	0.53 ± 0.16^a^
	21	4.62 ± 0.01^b,C^	0.97 ± 0.00^a,C^	39.12 ± 0.20^b,c,D^	0.69 ± 0.13^a^
	28	4.57 ± 0.02^b,C^	0.97 ± 0.00^a,B^	38.44 ± 0.55^a,b,A^	0.57 ± 0.14^a^
	56	4,60 ± 0.02^b,A^	0.97 ± 0.00^a,A^	38.12 ± 0.48^a,b,A^	0.52 ± 0.32^a^
*L. farciminis*	0	4.39 ± 0.04^a,C^	0.97 ± 0.00^a,B^	37.29 ± 0.11^a,B^	–
	7	4.47 ± 0.03^b,D^	0.97 ± 0.01^a,C^	36.78 ± 0.49^a,b,D^	0.37 ± 0.21^a^
	14	4.46 ± 0.02^b,C^	0.97 ± 0.00^a,C^	36.60 ± 0.50^a,b,A^	0.33 ± 0.14^a^
	21	4.50 ± 0.04^b,C^	0.97 ± 0.00^a,C^	35.58 ± 0.74^b,c,E^	0.58 ± 0.26^a^
	28	4.46 ± 0.03^b,C^	0.97 ± 0.00^a,B^	35.39 ± 0.40^b,c,B^	0.66 ± 0.17^a,b^
	56	4.47 ± 0.02^b,B^	0.97 ± 0.00^a,A^	34.33 ± 0.82^c,B^	1.16 ± 0.27^b^

*a, b, c, and d Mean values per treatment type with the same superscript are not statistically different (*P* > 0.05). A, B, C, D, and E Mean values per storage time with the same superscript are not statistically different (*P* > 0.05).*

As to the a_w_ of the pastes in the current study, the parameter did not vary significantly during storage, regardless of the preservative treatment and the storage time.

The color of the mealworm pastes was shown to be altered during storage as demonstrated by the browning index (BI) and the total color difference (ΔE). Browning can be a significant problem as it affects the visual quality of food products. As shown in Table 1, the browning index of the control pastes increased as storage time proceeded, which is not unexpected since mealworms are prone to browning upon processing (Van Campenhout et al., 2017). In the presence of sodium nitrite and sodium lactate, a significant (*p* < 0.05) reduction of the BI was observed during the first week of storage, but then it remained almost constant during the rest of the storage period. Using the same preservatives, De Smet et al. (2019) observed a similar evolution of the BI during storage, which might indicate that both preservatives have some antibrowning activity. Fermentation of the pastes did not present a definite trend in BI during storage but, as indicated by ΔE, the initial color of these pastes was preserved the best. In contrast, the most significant color changes were noticed in the control samples, where color differences up to 5.19 were recorded during storage. According to Wibowo et al. (2015), this differences can be visible. The total color difference of the pastes treated with additives remained below 1.75 during storage, which most likely is not visible.

### Microbial Counts During Storage

Microbiological analysis of freshly prepared mealworm pastes revealed the presence of LAB at 2.6 ± 0.1 log cfu/g, ENT at 2.6 ± 0.6 log cfu/g, and PSY at 1.5 ± 0.8 log cfu/g. Total aerobic and anaerobic plate counts ranged between 2.7 and 3.9 log cfu/g, which were in line with the findings of De Smet et al. (2019) for pastes prepared from steamed mealworms. At the start of the storage experiment, the total counts and LAB counts of the fermented samples were significantly (*p* < 0.05) higher than those of the unfermented samples due to the inoculation of the starter strains. Pastes inoculated with the starter culture Bactoferm^®^ F-LC had total counts and LAB counts of approximately 7.9 log cfu/g, compared to levels of 6.0 log cfu/g in samples inoculated with the starter culture *L. farciminis*. These levels, obtained after one week of fermentation, were similar to those reported in earlier studies on the fermentation of blanched mealworms (Borremans et al., 2018, 2019).

**FIGURE 1:**
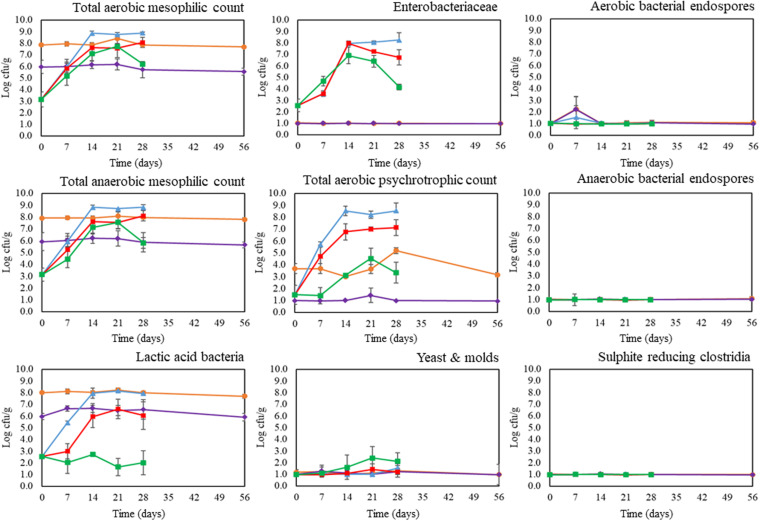
Microbial counts of control pastes (blue), pastes with sodium nitrate (red), pastes with sodium lactate (green), pastes fermented with Bactoferm^®^ F-LC (orange), and pastes fermented with *Lactobacillus farciminis* (purple) during storage at 4°C. Graphs show averages and standard deviations of three repetitions. The dotted horizontal line indicates the detection limit for the microbial counts.

Microbial changes in the mealworm pastes during refrigerated storage at 4°C are shown in Figure 1. Throughout the storage period, total counts and LAB counts of the fermented pastes remained relatively constant. In contrast, in control pastes and pastes containing a preservative, total counts and LAB counts (except the LAB counts of pastes with SL) increased dramatically (*p* < 0.05) during the first two weeks of storage, reaching values between 2.3 and 6.2 log cfu/g at day seven and between 6.7 and 9.1 log cfu/g at day 14. These counts are close to or above the level of 7.0 log cfu/g, which is considered as the microbiological acceptability limit for foods (Sperber and Doyle, 2009). Therefore, based on these counts, the microbiological shelf-lives of unfermented mealworm pastes would be between seven and 14 days, regardless of whether a preservative was added or not, and regardless of the type of preservative added. Using similar test conditions, De Smet et al. (2019) reached the same conclusions for pastes prepared from steamed mealworms. In this study, total counts of pastes with and without additives ranged between 2.8 and 5.6 log cfu/g at the start of the storage experiment, between 4.9 and 6.6 log cfu/g after one week of refrigerated storage and between 7.2 and 9.1 log cfu/g after two weeks of refrigerated storage.

As the total counts and the LAB counts of fermented samples were high due to the inoculation of the starter strains, other microbial counts like PSY, ENT, Y&M, SRC, and spore counts were chosen as factors determining the microbiological shelf-lives of fermented pastes. The presence of some psychrotrophs and Enterobacteriaceae in the mealworm pastes at the start of storage might involve a risk of spoilage in a chilled environment (Hwang and Tamplin, 2005; Martínez et al., 2007). Unfermented samples experienced a significant (*p* < 0.05) increase in both microbial counts during storage. Moreover, between one and two weeks of storage, the number of ENT exceeded their maximum limit of 5.0 log cfu/g for insects and insect-based products as proposed by the Federal Agency for Safety of the Food Chain (FASFC, 2018). In contrast, during storage of fermented samples, the acidified conditions completely inhibited the growth of ENT (<1.0 log cfu/g), whereas the levels of PSY remained (relatively) stable at acceptable levels (<5.2 log cfu/g). Note hereby that, both at the start as during storage, the PSY counts of the pastes fermented with the starter culture Bactoferm^®^ F-LC were significant (*p* < 0.05) higher than those of the pastes fermented with the starter culture *L. farciminis*. This can be explained, and was also confirmed by microbiological analysis of the starter cultures, by the fact that one or more strains of the mixed culture Bactoferm^®^ F-LC can grow at refrigerated temperatures. Edible insects are also prone to spoilage caused by yeast and molds. During storage, the highest numbers of yeast and molds, up to 3.0 log cfu/g, were found in the pastes with the preservative sodium lactate. These counts were still below the minimum limit of 3.7 log cfu/g for fungi (FASFC, 2018). Finally, no or limited growth in SRC and aerobic and anaerobic bacterial endospores were observed in all samples throughout the storage period.

*Bacillus cereus*, *Salmonella* spp., and *Listeria monocytogenes* were not detected in any of the mealworm samples tested (<100 cfu/g, absence in 25 grams, and absence in 25 grams, respectively). The fact that microbial counts increased substantially in unfermented samples can be an indication that, if certain pathogens would be present in the starting material, they may grow as well and potentially cause safety problems. Whether fermentation can prevent this, still needs to be confirmed, for instance via challenge tests. Overall, the microbial counts of the fermented pastes in this study were below the permissible limits, which indicated that the product was safe for consumption, during the whole test period, i.e., eight weeks.

### Lipid Oxidation During Storage

Mealworms are rich in fats (10–30% DM) and contain high amounts of unsaturated fatty acids (70–75%), which makes them vulnerable to lipid oxidation during storage (Lenaerts et al., 2018; Kröncke et al., 2019). The peroxide and *p*-anisidine values, used as an index for primary and secondary lipid oxidation, respectively, are shown in Table 2. Similar as in the mealworm pastes prepared from steamed larvae (De Smet et al., 2019), no primary oxidation occurred in the pastes investigated in this study. During storage, the peroxide values remained below the detection limit of 0.5 meq. O_2_/kg fat. However, primary oxidation products are very unstable and decompose rapidly into secondary oxidation products that are responsible for sensory deterioration (Domínguez et al., 2019). Thus, when evaluating the fat quality, both parameters have to be considered. As shown in Table 2, *p*-anisidine values greater than zero were found for all samples. The *p*-anisidine values of the fermented pastes were higher than those of the unfermented pastes at the start of the storage period indicating that oxidation already occurred during fermentation. While their values increased toward the end of the experiment, *p*-anisidine values of the unfermented samples decreased. Despite these differences, all anisidine values were far below the EFSA criterion for fish oil (*p*-anisidine < 20), which is often used as a reference for edible insects. So it can be concluded that the oxidation status of all the pastes were acceptable until the end of the storage period, regardless of the preservative treatment applied.

**TABLE 2:** Peroxide and *p*-anisidine values of pastes prepared from blanched mealworms without additive (Control), with the preservatives sodium nitrite or sodium lactate or fermented with the starter cultures Bactoferm^®^ F-LC or *Lactobacillus farciminis* and stored at 4°C for 28 (unfermented pastes) or 56 days or 8 weeks (fermented pastes).

Sample	Sampling day	Peroxide value (meq.O_2_/kg fat)	*p*-anisidine value (–)
Control	0	<LOD	1.39 ± 0.16^a,A^
	28	<LOD	1.05 ± 0.01^b,A,B^
Sodium nitrite	0	<LOD	1.39 ± 0.16^a,A^
	28	<LOD	0.86 ± 0.21^b,A^
Sodium lactate	0	<LOD	1.39 ± 0.16^a,A^
	28	<LOD	0.72 ± 0.20^b,A^
Bactoferm^®^ F-LC	0	<LOD	1.99 ± 0.17^a,B^
	28	<LOD	1.84 ± 0.27^a,C^
	56	<LOD	2.76 ± 0.24^b,A^
*L. farciminis*	0	<LOD	1.85 ± 0.21^a,B^
	28	<LOD	1.76 ± 0.50^a,B,C^
	56	<LOD	2.75 ± 0.79^a,A^

*LOD = Limit of detection (0.5 meq.O_2_/kg fat). a, b Mean values per treatment with the same superscript are not statistically different (*P* > 0.05). A, B, C Mean values per storage time with the same superscript are not statistically different (*P* > 0.05). Values are means of three replicates ± standard deviation.*

### Cost-Effectiveness and Application Potential

While fermentation shows to be a promising conservation strategy for mealworm pastes, a number of aspects still need further research to assess the cost-effectiveness and application potential of fermented pastes. In the first place, future research on mealworm fermentation and storage of fermented pastes also needs to consider the impact on taste and consumer acceptance. Having a pH of around 4.5, the fermented product likely contains a large amount of lactic acid which causes a favorable or unfavorable taste for animals and humans. Whether fermentation can also be applied for other insect species that are reared at industrial scale, also remains to be investigated. If the taste is (at least) acceptable, then a range of applications in feed, petfood and food industry can be envisaged. Currently, when insects are used in a feed formulation for farm animals, mealworms (and other insects) are dried (or frozen and then dried), because it is the only conservation technology known so far and applied in industry to provide a shelf life long enough to set up a proper logistic chain. However, freezing, freeze drying, and oven drying are known to imply a substantial cost price, mainly due to energy consumption (Lenaerts et al., 2018). Feed producers are able to include up to 10% of liquid ingredients in their dry feed formulas, which makes them interested in insects as feed ingredients that are stabilized in other (and more cost-efficient) ways than drying (Heutink, ForFarmers, personal communication). If their taste is accepted by pets, fermented insect pastes may well find an application as palatant or digest to be sprayed on kibbles or included in wet foods. Also, petfood manufacturers are increasingly looking at opportunities to claim probiotic effects for their wet and dry formulas (Anonymous, 2016; Coman et al., 2019), which could be an asset of integrating fermented insects as well. In the same way as for animal feed, in the food industry fermented (and hence stabilized) insect pastes may likely be incorporated in foods as alternative to dried insects, or new functional ingredients may be developed from fermented insects.

## Conclusion

The addition of a sodium nitrite and a sodium lactate preservative at their maximally allowed concentration to pastes prepared from blanched mealworms could not prevent microbial spoilage during refrigerated storage. In contrast, lactic acid fermentation of the pastes with Bactoferm^®^ F-LC or *L. farciminis* avoided microbial spoilage during the whole test period of eight weeks Typical food pathogens such as *Bacillus cereus*, *Salmonella* spp., and *Listeria monocytogenes* were not detected in any of the mealworm samples tested. Future research with mealworms artificially contaminated with food pathogens has to elucidate whether fermentation can also prevent outgrowth of those pathogens. Microbial growth occurred at a faster rate than lipid oxidation and was the major determinant of shelf life. This study demonstrates that lactic acid fermentation is a valuable preservation technique for mealworm pastes.

## Data Availability Statement

The original contributions presented in the study are included in the article, further inquiries can be directed to the corresponding author.

## Author Contributions

AB designed and performed the experiments, processed the data, and drafted the manuscript with input from all authors. RS assisted with the fat oxidation measurements. LV supervised the project, responsible for the financing and edited and proofread the text. All authors contributed to the article and approved the submitted version.

## Conflict of Interest

The authors declare that the research was conducted in the absence of any commercial or financial relationships that could be construed as a potential conflict of interest.
